# The impact of adverse childhood experiences on cosmetic surgery addiction in cosmetic surgery patients

**DOI:** 10.3389/fpubh.2025.1682796

**Published:** 2025-09-25

**Authors:** Zhujing Li, Qingjiang Huang, Zhenghui Li, Fengwen Yue, Ying Lei, Hongyan Liu

**Affiliations:** Beijing Anzhen Nanchong Hospital of Capital Medical University, Nanchong Central Hospital, Nanchong, China

**Keywords:** cosmetic surgery patients, adverse childhood experiences, cosmetic surgery addiction, insecure attachment, self-alienation

## Abstract

**Background:**

Previous studies have found that cosmetic surgery patients often exhibit repetitive and compulsive behaviors associated with cosmetic surgery addiction. Adverse childhood experiences have been linked to various psychological and behavioral issues; however, their relationship with cosmetic surgery addiction has not been explored. This study aims to investigate the impact of adverse childhood experiences on cosmetic surgery addiction and its underlying mechanisms, including the mediating roles of insecure attachment and self-alienation, in cosmetic surgery patients. These mediators were assessed based on attachment theory and existential psychology, which posit that childhood trauma disrupts secure relationships and self-integration, potentially driving compensatory addictive behaviors.

**Methods:**

We employed a cross-sectional design and recruited 605 cosmetic surgery patients from four tertiary grade A hospitals in Sichuan, China, between April 25th to May 29th 2025. Participants completed questionnaires assessing adverse childhood experiences, insecure attachment, self-alienation, and cosmetic surgery addiction. We tested both direct and indirect effects of adverse childhood experiences on cosmetic surgery addiction using Structural Equation Modeling and Process Model 6.

**Results:**

Our findings revealed that adverse childhood experiences significantly positively predicted cosmetic surgery addiction in cosmetic surgery patients (*β* = 0.194, *p* < 0.001, 95% CI = [0.107, 0.281]). Furthermore, insecure attachment (Indirect effects: *β* = 0.079, SE = 0.036, 95%CI = [0.021, 0.152]) and self-alienation (Indirect effects: *β* = 0.066 SE = 0.031, 95%CI = [0.011, 0.133]) exacerbated the tendency toward cosmetic surgery addiction among patients with adverse childhood experiences (Chain effect: *β* = 0.033, SE = 0.013, 95%CI = [0.006, 0.058]).

**Conclusion:**

This study highlights the importance of interventions targeting insecure attachment and self-alienation to reduce the risk of cosmetic surgery addiction in patients with adverse childhood experiences. These findings further emphasize the importance of addressing psychological mechanisms in the care of cosmetic surgery patients to promote healthier psychological outcomes.

## 1 Introduction

Cosmetic surgery refers to the medical procedures aimed at improving and restoring patients’ physical appearance (1), primarily to enhance attractiveness, correct defects caused by accidents or illnesses, and fulfill individual aesthetic aspirations (2). For this study, cosmetic surgery is defined as invasive surgical procedures involving incisions, tissue manipulation, or anesthesia to enhance aesthetic appearance (e.g., rhinoplasty, breast augmentation, liposuction, hair transplantation), excluding non-surgical or minimally invasive interventions such as dermal fillers, Botox injections, chemical peels, or laser therapies. This distinction aligns with prior research focusing on addictive patterns in surgical contexts (3) and is reflected in the demographic data, which categorizes surgical sites. This has led to a broad audience for cosmetic surgery, encompassing individuals of various ages, genders, and social backgrounds (4, 5). While many patients undergo cosmetic surgery for reasonable aesthetic or functional needs (6), some develop an addiction to repeated procedures, exhibiting signs of cosmetic surgery addiction (7). Cosmetic surgery addiction is characterized by an excessive reliance on surgical interventions, an obsessive focus on perceived flaws in one’s appearance, and a compulsive pursuit of further surgeries, often resulting in psychological distress and a diminished quality of life (8, 9). Previous studies have identified that societal standards, excessive media promotion, professional requirements, and positive feedback from previous surgeries can reinforce addictive behaviors (10). For instance, Pearlman et al. (11) found that the improvement and restoration of appearance through cosmetic surgery can enhance confidence and social recognition, thereby reinforcing the desire to undergo further procedures. Although prior studies have explored certain psychological mechanisms underlying cosmetic surgery addiction, such as body dissatisfaction, social media influence, and reinforcement from positive outcomes (12–14), gaps remain in understanding how early-life trauma, like adverse childhood experiences, contributes via relational and existential pathways. For instance, while Ateq et al. (15) and Pinto et al. (16) have examined obsessive traits and motivational factors, the roles of insecure attachment and self-alienation as mediators have been underexplored, particularly in non-Western populations. Recent research has also confirmed that cosmetic surgery addiction can lead to severe financial burdens and health risks (17). This study addresses these gaps by focusing on the psychological factors underlying addictive behaviors in cosmetic surgery patients, which holds significant practical implications for optimizing clinical intervention strategies, preventing potential psychological issues, and promoting societal understanding of cosmetic surgery addiction.

Adverse childhood experiences refer to various forms of negative events experienced during childhood, including domestic violence, emotional abuse, physical abuse, and emotional neglect (18, 19). These experiences not only lead to severe psychological problems, such as anxiety, depression, and post-traumatic stress disorder (20, 21), but also increase the risk of various physical illnesses, including cardiovascular diseases, diabetes, and immunological disorders (22, 23). Consequently, many researchers have begun to investigate psychological interventions for individuals with adverse childhood experiences, proposing measures such as emotion regulation (24) and socio-economic interventions (25). Notably, Hays-Grudo et al. (26) proposed that interventions promoting secure attachment through Attachment Biobehavioral Catch-up strategies can effectively reduce the intergenerational cycle of adverse childhood experiences and addictive behaviors. However, their study did not examine the relationship between adverse childhood experiences and addictive behaviors. In other words, whether adverse childhood experiences serve as a predictive factor for cosmetic surgery addiction among patients has not been explored. Adverse childhood experiences may lead to issues such as low self-esteem, body dissatisfaction, and identity confusion (27). According to the psychological compensation theory (28), unmet psychological needs in childhood may be compensated for in adulthood through external means (29), with cosmetic surgery becoming a tool for self-worth enhancement. For example, children who are consistently mocked by peers for their appearance may internalize these negative evaluations, leading to extreme dissatisfaction with their appearance (30). In adulthood, they are more likely to seek change through cosmetic surgery, and upon receiving positive feedback from such procedures, they may become trapped in a cycle of cosmetic surgery addiction. Based on the above analysis, we propose hypothesis H1: Adverse childhood experiences positively predict cosmetic surgery addiction in patients.

However, what are the internal mechanisms linking adverse childhood experiences and cosmetic surgery addiction? Based on attachment theory (31), adverse childhood experiences may impair the formation of an early secure base, increasing the risk of insecure attachment (32). However, during development, individuals with insecure attachment, who fail to establish stable and secure attachment relationships, lack a sense of safety and self-worth (33–35). As they enter adulthood and face societal pressures emphasizing physical appearance, the distortions in self-perception and emotional instability caused by insecure attachment make them more likely to view physical appearance as a key tool for gaining recognition and avoiding rejection. Consequently, they may transform repetitive cosmetic surgeries into a pathological strategy for emotional regulation and relationship maintenance. Specifically, individuals with anxious attachment may constantly adjust their appearance to alleviate their extreme fear of abandonment and seek continuous affirmation (36). In contrast, those with avoidant attachment may view physical alteration as a source of control, replacing the emotional intimacy they find difficult to establish. More importantly, the temporary positive feedback from cosmetic surgeries can alleviate the deep-seated pain associated with insecure attachment (37), such as feelings of shame and emptiness, forming a strong negative reinforcement cycle that closely resembles the neurological mechanisms of substance or behavioral addiction (38). The temporary relief following each surgery reinforces the dependency on cosmetic procedures (39), ultimately leading to increased tolerance and withdrawal symptoms, thereby strengthening the cycle of cosmetic surgery addiction. Thus, insecure attachment not only serves as a critical psychological consequence of adverse childhood experiences but also constitutes the core psychological driving mechanism through which dissatisfaction with one’s appearance is transformed into compulsive and addictive cosmetic behaviors, profoundly influencing the trajectory of development from childhood trauma to pathological bodily interventions in adulthood. Based on the above analysis, we propose hypothesis H2: Insecure attachment plays a significant mediating role in the relationship between adverse childhood experiences and cosmetic surgery addiction.

Previous studies have widely confirmed that adverse childhood experiences can severely impair the development of a healthy sense of self (40, 41). According to the theory of the “true self” and “false self” (42), individuals who repeatedly experience trauma or neglect are often forced to suppress their true emotions and needs to adapt to adverse environments, developing a highly functional “false self”. The disconnect between the individual and their true self is referred to as self-alienation (43, 44). This self-alienation manifests as chronic emotional numbing, confusion in internal experiences, distorted self-worth, and difficulty identifying or trusting one’s internal states, forming core identity disturbances (45, 46). When such individuals enter adulthood, physical appearance, as a relatively controllable and externally visible aspect of self-representation (47), becomes a central pathway for rebuilding self-perception and bridging the gap between the true self and the ideal self. Cosmetic surgery becomes a compulsive strategy to alleviate the unbearable sense of self-alienation, temporarily gaining a sense of control and external recognition. However, since the fundamental issue of self-alienation remains unresolved, the temporary satisfaction provided by each cosmetic procedure quickly fades, forcing individuals into a “need-behavior-temporary relief-need reemergence” cycle of addiction, ultimately leading to excessive or repetitive addictive patterns of surgery. Based on the above analysis, we propose hypothesis H3: Self-alienation plays a significant mediating role in the relationship between adverse childhood experiences and cosmetic surgery addiction.

Insecure attachment significantly exacerbates the development of self-alienation. Individuals with anxious attachment, due to their overemphasis on others’ evaluations, often closely link their self-worth to external standards, leading to a severe disconnect between their true and ideal selves (37). In contrast, those with avoidant attachment may actively suppress their inner experiences to defend against vulnerability, resulting in a deep disconnection from their true feelings (48). This state of self-alienation prevents individuals from constructing a stable identity based on their internal needs, leading them to rely heavily on external, visible, and controllable traits to define themselves and seek temporary confirmation of their worth (49, 50). This profound self-alienation significantly influences addictive cosmetic surgery behavior. Therefore, adverse childhood experiences impair the ability of cosmetic surgery patients to establish secure emotional connections, and this deficit further hinders the development of a healthy sense of self. Consequently, individuals are compelled to seek existence and self-worth through extreme physical transformations, leading to an uncontrollable dependency on cosmetic surgeries. Based on the above analysis, we propose hypothesis H4: Insecure attachment and self-alienation play a significant chain-mediating role in the relationship between adverse childhood experiences and cosmetic surgery addiction.

For clarity, Table 1 summarizes the key theories referenced in this study, including brief definitions, foundational references, and their relevance to our investigation of adverse childhood experiences (ACEs) and cosmetic surgery addiction (CSA).

**Table 1:** Summary of theoretical frameworks.

Theory	Definition	Key references	Relevance to this study
Psychological compensation theory	A process where individuals subconsciously develop strengths or behaviors in one area to offset perceived deficiencies or inadequacies in another, often to restore self-esteem or adapt to unmet needs. This can manifest as overachievement or substitution strategies to mitigate feelings of inferiority.	Bäckman and Dixon (28); Adler—foundational in individual psychology, emphasizing compensation for organ inferiority or psychological deficits.	ACEs often lead to low self-esteem and body dissatisfaction (27). In this study, cosmetic surgery serves as a compensatory mechanism, where patients seek external validation through repeated procedures to address unresolved childhood trauma, directly linking to the positive prediction of CSA (H1).
Attachment theory	Posits that early interactions with caregivers form an internal working model of relationships, influencing emotional bonds and security throughout life. Secure attachment fosters trust and self-regulation, while insecure types (anxious or avoidant) result from inconsistent caregiving, leading to relational anxiety, avoidance, or maladaptive coping in adulthood.	Bowlby (34, 35, 56)—Bowlby emphasized the evolutionary “attachment behavioral system” for proximity-seeking; relevant to adult behaviors like seeking reassurance or emotional distancing.	ACEs disrupt secure attachment formation, fostering insecure attachment that mediates the path to CSA (H2). Patients may use cosmetic surgery as a pathological strategy for emotional regulation and gaining approval, amplifying addictive cycles due to unmet relational needs.
True self and false self-theory	Describes the “true self” as an authentic, spontaneous sense of being alive and integrated, emerging from supportive environments. The “false self” is a defensive facade developed to comply with external demands, protecting the vulnerable true self but leading to disconnection, inauthenticity, and emotional suppression when environments fail (e.g., via neglect).	Winnicott (59)—Winnicott viewed this as a response to “environmental failures,” where the false self masks the true self to ensure survival.	ACEs force suppression of the true self, resulting in self-alienation (H3). In CSA, repeated surgeries represent futile attempts to bridge this disconnect, reconstructing an external “ideal self” to alleviate identity confusion and existential distress.
Ecological systems theory	Views human development as influenced by nested environmental systems: microsystem (immediate interactions, e.g., family), mesosystem (interconnections), exosystem (indirect influences), macrosystem (cultural values), and chronosystem (time-based changes). Emphasizes bidirectional interactions between the individual and these layers.	Bronfenbrenner (83)—Bronfenbrenner highlighted how microsystem disruptions (e.g., family trauma) ripple across systems, shaping long-term outcomes.	ACEs in the microsystem (e.g., abusive family) disrupt development, leading to adult maladaptive behaviors like CSA. This theory justifies examining ACEs’ broad ecological impact, including how societal beauty standards (macrosystem) exacerbate addiction in trauma survivors.
Self-alienation theory	Refers to a psychological state of disconnection from one’s authentic self or society, involving emotional numbing, identity fragmentation, and hostility toward one’s inner experiences. Often stems from trauma or social structures, leading to existential ills and maladaptive coping.	Winnicott (59); extensions from Hegel/Marx on alienation. In psychology, it builds on existential and social theories, viewing alienation as a separation between self and other/true needs.	Integrates with true/false self-concepts; ACEs via insecure attachment exacerbate self-alienation, driving CSA as a compulsive external fix (H3, H4). This chain mediation highlights how unresolved alienation perpetuates addictive cycles in cosmetic patients.

**Figure 1:**
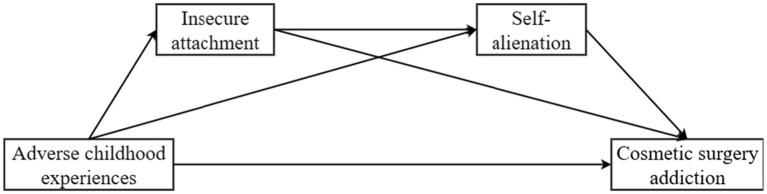
Research framework diagram.

The theoretical framework of the research model is shown in Figure 1.

## 2 Methods

### 2.1 Study design and setting

This study utilized a cross-sectional design to examine the relationships among adverse childhood experiences, insecure attachment, self-alienation, and cosmetic surgery addiction. Data collection occurred at four tertiary Grade A hospitals in Sichuan Province, China, from April 25th to May 29th, 2025. These hospitals were selected for their high volume of cosmetic surgery procedures and diverse patient demographics, ensuring a representative sample from urban and rural areas.

### 2.2 Participants

#### 2.2.1 Recruitment process

To validate the aforementioned hypotheses, this study conducted participant recruitment from April 25th to May 29th 2025 at four tertiary class A hospitals in Sichuan Province. We employed a convenience sampling method, recruiting consecutive patients who visited the plastic surgery departments during the study period and met the inclusion criteria. This approach was chosen for its feasibility in a clinical setting, allowing access to a large pool of eligible participants while minimizing selection bias through systematic inclusion of all qualifying individuals. Specifically, we contacted the directors of the plastic surgery departments and submitted our research plan for their review. Upon obtaining approval from the department director, we conducted data collection in both the inpatient and outpatient departments of the hospitals. All participants were fully informed of the study’s purpose, procedures, and potential risks. Written informed consent was obtained from each participant before they completed the questionnaire. This study was approved by the ethics committee of the Nanchong Central Hospital and adhered to all relevant ethical guidelines.

#### 2.2.2 Inclusion and exclusion criteria

Inclusion criteria: (1) Patients did not exhibit significant mental disabilities or speech impairments. (2) Patients were diagnosed with cosmetic surgery intentions by specialized physicians. (3) Patients had not participated in similar studies in the past week. (4) Patients provided written informed consent. We included “Patients had not participated in similar studies in the past week” as an inclusion criterion, which prevents participant fatigue and minimizes bias in self-reported data with repeated exposures. On the other hand, patients frequently participate in similar studies, and they may have a learning effect whose behavior or response may not be the true response of the subject. In this study, cosmetic surgery intentions to undergo cosmetic surgery were selected as the inclusion criteria and assessed by a hospital board-certified plastic surgeon to confirm aesthetic intentions and propensity for cosmetic surgery. The assessment modality consisted of a structured clinical interview that included motivation, expectations, and body image questions raised by following a standard preoperative assessment protocol. Specialist doctors recorded confirmation in the medical record to ensure that cases with non-cosmetic surgery intentions were excluded and included in the study, thereby improving the accuracy of the study.

Exclusion criteria: (1) Patients suffered from severe mental illnesses or lacked normal speech capabilities. (2) Patients had participated in cross-sectional studies in the previous week (to minimize participant fatigue, recall bias, or priming effects that could influence responses in self-report measures). (3) Patients whose native language was not Chinese, leading to difficulty in understanding the questionnaire. (4) Questionnaires exhibited strong consistency bias in responses (assessed via visual inspection for uniform patterns, such as all items scored identically or failing reverse-scored consistency checks, indicating potential inattentive or dishonest responding). (5) Patients refused to sign the informed consent form or withdrew from the survey midway.

#### 2.2.3 Sample size calculation

This study used F-test analysis via G*Power software to calculate the minimum required sample size. The statistical test selected was “Linear multiple regression: Fixed model, R^2^ deviation from zero,” with an effect size (f^2^) of 0.15, a significance level (*α*) of 0.05, and a statistical power (1-*β*) of 0.95. The analysis indicated that the minimum sample size required was 119.

This study first considered the valid return rate of the questionnaires. During the survey process, participants might provide responses with excessively short completion times, exhibit patterned answering patterns, omit items, or display logical inconsistencies—all of which necessitated an increase in sample size. Additionally, during the data cleaning phase, it was essential to remove outliers, missing values, and responses that failed attention-check questions, further underscoring the need for a larger sample. Finally, consistent with findings from Cao et al. (51), merely meeting the minimum sample size requirement may lead to coincidental or unreliable results. Data fluctuations could also affect significance levels (52). A larger sample size allows for more accurate estimation of effect sizes and enhances the robustness of the findings. Therefore, a total of 640 questionnaires were distributed in this study.

#### 2.2.4 Demographic

To meet the minimum sample size requirement and account for potential invalid samples, this study distributed 640 questionnaires. During the data cleaning phase, 13 paper questionnaires were excluded due to damage, 9 participants withdrew midway, and 13 questionnaires exhibited strong response consistency bias. Ultimately, 605 valid questionnaires were included, yielding an effective response rate of 94.53%.

### 2.3 Operational definitions and scale alignment

In this study, the four key variables were operationalized based on established theoretical frameworks and measured using validated scales adapted for cultural relevance. We define each construct and explain its alignment with the selected scale, including how sub-dimensions correspond to the conceptual definitions.

Adverse Childhood Experiences: Defined as a range of traumatic events occurring before age 18, including abuse (emotional, physical, sexual), neglect (emotional, physical), and household dysfunction (e.g., parental separation, substance abuse, mental illness), which disrupt healthy development and increase vulnerability to psychopathology (18, 53). This aligns with the Adverse Childhood Experiences List (54) [Chinese validation by Zhao et al. (55)], a 14-item scale without formal sub-dimensions but categorically covering abuse (e.g., items on physical/emotional harm), neglect (e.g., items on unmet needs), and dysfunction (e.g., items on family instability). Items are summed for a total score (14–70), reflecting cumulative exposure, which matches our conceptual focus on severity and breadth of trauma.

Insecure Attachment: Conceptualized as maladaptive relational patterns stemming from early caregiving failures, characterized by anxiety (fear of abandonment, excessive reassurance-seeking) and avoidance (emotional distancing, discomfort with intimacy), leading to interpersonal difficulties and self-regulation deficits (34, 35, 56). This is measured by the Experiences in Close Relationships-Revised (ECR-R) scale (57) [Chinese validation by Xing and Xu (58)], with 36 items divided into two sub-dimensions: Anxiety (18 items assessing hyperactivation of attachment system) and Avoidance (18 items assessing deactivation). Subscale averages contribute to a total score (36–180), with higher values indicating greater insecurity, directly corresponding to our definition by capturing both emotional and behavioral manifestations.

Self-Alienation: Defined as a profound disconnection from one’s authentic self, involving emotional numbing, identity confusion, and suppression of true needs to adapt to external demands, often resulting from trauma and leading to existential distress (44, 59). The adapted Self-Alienation Scale (60) comprises 27 items without explicit sub-dimensions but thematically encompassing emotional detachment (e.g., items on feeling numb to inner experiences), identity fragmentation (e.g., items on confusion about true self), and adaptive suppression (e.g., items on masking needs). Total summed score (27–135) reflects overall alienation severity, aligning with our conceptual emphasis on self-disintegration as a mediator.

Cosmetic Surgery Addiction: Operationalized as a behavioral addiction involving compulsive pursuit of procedures, tolerance (need for more surgeries), withdrawal (distress without intervention), and interference with life functioning, driven by obsessive body dissatisfaction (61). The Cosmetic Surgery Addiction Scale includes 24 items across four sub-dimensions: Tolerance-Dependence (e.g., items on escalating need for surgeries), Life Interference (e.g., items on social/financial disruption), Positive Expectations (e.g., items on anticipated benefits reinforcing behavior), and Pathological Obsession (e.g., items on rumination about flaws). Subscale averages yield a total score (24–120), precisely matching our definition by quantifying addictive cycles.

These alignments ensure construct validity, with sub-dimensions providing granular insights into mechanisms.

### 2.4 Translation and adaptation procedure

For scales originally in English and Korean (Cosmetic Surgery Addiction Scale and Self-Alienation Scale), we employed Brislin (62) back-translation method to ensure linguistic and cultural equivalence. The process involved: (1) Forward translation from English to Chinese by a bilingual expert in psychology and cosmetic medicine; (2) Independent back-translation to English by a second bilingual translator unfamiliar with the original scale; (3) Comparison of the back-translated version with the original by the research team to identify discrepancies in meaning, wording, or cultural nuances; (4) Resolution of issues through iterative discussions and consensus, involving a third expert if needed; and (5) Pilot testing on a small sample (n = 30) of cosmetic surgery patients to assess comprehensibility and cultural fit, with minor refinements for idiomatic expression. This rigorous procedure minimized translation biases and enhanced cross-cultural validity. Psychometric properties were further established through confirmatory factor analysis (CFA) in AMOS 29.0, reporting goodness-of-fit indices (GFI > 0.90, RMSEA < 0.08), and internal consistency via Cronbach’s *α* (> 0.90 for all scales), confirming reliability in the Chinese context.

### 2.5 Measurement tools

All measurement items are shown in Appendix.

#### 2.5.1 Cosmetic surgery addiction scale

This study used the Cosmetic Surgery Addiction Scale (CSAS) initially developed by Lim (61). The scale consists of 24 items across four dimensions: tolerance-dependence, life interference, positive expectations, and pathological obsession. Although the scale has been primarily used in Korean studies, this research employed back-translation to enhance cultural adaptability (62). Each item was rated on a 5-point Likert scale, ranging from “1 = Strongly Disagree” to “5 = Strongly Agree,” with higher scores indicating greater tendencies toward cosmetic surgery addiction. Subscale scores were calculated by averaging relevant items, and the total score ranged from 24 to 120. Using AMOS 29.0, the model fit was assessed, yielding satisfactory results (GFI = 0.918, AGFI = 0.869, RMSEA = 0.082, CFI = 0.937, TLI = 0.911). Reliability analysis also revealed strong internal consistency (Cronbach’s *α* = 0.941).

#### 2.5.2 Insecure attachment scale

This study assessed insecure attachment (anxiety and avoidance dimensions) among cosmetic surgery patients using the adult attachment scale developed by Fraley et al. (57). The scale was translated into Chinese and validated by Xing and Xu (58) for cultural adaptability. Responses were recorded on a 5-point Likert scale (1 = Strongly Disagree to 5 = Strongly Agree). Higher scores on the avoidance dimension indicated a greater tendency to avoid emotional intimacy and seek external help, while higher scores on the anxiety dimension reflected heightened concerns about relationship maintenance. The scale has 36 items (18 per dimension); scoring involved averaging items per subscale, with total scores ranging from 180 to 36 (higher indicating greater insecurity). Both the model fit (GFI = 0.858, AGFI = 0.839, RMSEA = 0.051, CFI = 0.914, TLI = 0.909) and reliability (Cronbach’s *α* = 0.964) demonstrated excellent psychometric properties.

#### 2.5.3 Self-alienation scale

The self-alienation scale used in this study was adapted from the Self-Alienation Scale developed by Nishiyama et al. (60). The scale consists of 27 items, and back-translation was applied to ensure cultural equivalence (62). Each item was measured using a 5-point Likert scale ranging from “1 = Strongly Disagree” to “5 = Strongly Agree,” with higher scores indicating greater feelings of self-alienation. Scoring involved summing all items and the total score ranged from 27 to 135. The model fit analysis conducted via AMOS 29.0 showed satisfactory results (GFI = 0.904, AGFI = 0.884, RMSEA = 0.051, CFI = 0.928, TLI = 0.919), and the internal consistency was strong (Cronbach’s *α* = 0.942).

#### 2.5.4 Adverse childhood experiences scale

Adverse Childhood Experiences was assessed using the Adverse Childhood Experiences List developed by Finkelhor et al. (54), which includes 14 items. The scale was translated into Chinese and validated by Zhao et al. (55) for cultural adaptability. Responses were collected on a 5-point Likert scale, with higher scores indicating more severe adverse childhood experiences. Scoring involved summing all items (no reverse-coding needed); total scores ranged from 14 to 70. Both the model fit (GFI = 0.961, AGFI = 0.947, RMSEA = 0.045, CFI = 0.979, TLI = 0.975) and reliability (Cronbach’s *α* = 0.936) demonstrated strong psychometric properties.

### 2.6 Data analysis

The analysis process began with model fit testing for each variable using AMOS 29.0 and reliability testing using SPSS 27.0. Subsequent analyses included common method bias testing, normality testing, descriptive statistics, and correlation analysis. Finally, we used the SPSS Macro Process Model to examine the chain mediating effects of insecure attachment and self-alienation. Adverse childhood experiences was treated as the independent variable, self-alienation and insecure attachment as mediators, and cosmetic surgery addiction as the dependent variable. A bias-corrected bootstrapping method was applied to test the significance of the mediating effects, with 5,000 bootstrap iterations. A 95% confidence interval that did not include zero was considered statistically significant (*p* < 0.05).

## 3 Result

### 3.1 Descriptive statistics of participants

Among the valid samples, 123 participants (20.3%) were male, and 482 participants (79.7%) were female. The educational background of the participants was as follows: 25 (4.1%) had primary school education or below, 122 (20.2%) completed junior high school, 182 (30.1%) completed senior high school, and 228 (37.7%) held a bachelor’s degree or higher. The average age of the participants was 27.93 ± 5.846 years. Detailed demographic information is presented in Table 2.

**Table 2:** Summary table of demographic information.

Variables	Items	Number	Proportion
Gender	Male	123	20.3%
	Female	482	79.7%
Education background	Primary School	25	4.1%
	Junior High School	122	20.2%
	Senior High School / College	182	30.1%
	Undergraduate	228	37.7%
	Master’s Degree	38	6.3%
	Doctoral Degree	10	1.7%
Marital status	Unmarried	305	50.4%
	Married	166	27.4%
	Divorced	110	18.2%
	Widowed	24	4.0%
Residence	Urban	375	62.0%
	Rural	230	38.0%
Income level	≤ 1,000 yuan	23	3.8%
	1,001–3,000 yuan	122	20.2%
	3,001–5,000 yuan	217	35.9%
	5,001–8,000 yuan	193	31.9%
	Above 8,000 yuan	50	8.3%
Cosmetic surgery site	Face	172	28.4%
	Chest	195	32.2%
	Liposuction	162	26.8%
	Hair grafting	76	12.6%
Number of cosmetic surgery	3 times or less	280	46.3%
	4 to 5 times	258	42.6%
	6 times or more	67	11.1%

### 3.2 Common method bias analysis

Given that the questionnaires were completed in a single session, used the same scale format, and could be influenced by social desirability bias, the potential for common method bias was a concern (63). To minimize this issue, a fully anonymous data collection approach was employed. Additionally, Harman’s single-factor test was conducted by performing an unrotated exploratory factor analysis on all self-report measures. The first factor explained 27.181% of the variance, which was below the 40% threshold. This indicated that common method bias did not significantly influence the results of this study.

### 3.3 Descriptive statistics and correlation analysis

This study performed descriptive statistics and correlation analyses on adverse childhood experiences, self-alienation, insecure attachment, and cosmetic surgery addiction. As shown in Table 3, the skewness (0.036 to 0.293) and kurtosis (−0.329 to −0.097) of the study variables fell within the thresholds proposed by Kline (64) (skewness < ±3, kurtosis < ±8), indicating that the data were normally distributed.

**Table 3:** Correlation analysis and descriptive statistics of all variables.

Variables	M	SD	Skewness	Kurtosis	1	2	3	4
1. Adverse childhood experiences	2.970	0.873	0.036	−0.329	1			
2. Insecure attachment	3.282	0.765	0.293	−0.097	0.445***	1		
3. Self-alienation	3.204	0.736	0.175	−0.132	0.568***	0.591***	1	
4. Cosmetic surgery addiction	3.267	0.861	0.175	−0.272	0.376***	0.372***	0.394***	1

M, Mean; SD, Standard Deviation; ***p < 0.001.

The correlation analysis revealed the following significant relationships: Adverse childhood experiences was significantly and positively correlated with insecure attachment (*r* = 0.445, *p* < 0.001; moderate correlation). Adverse childhood experiences was significantly and positively correlated with self-alienation (*r* = 0.568, *p* < 0.001; strong correlation). Adverse childhood experiences was significantly and positively correlated with cosmetic surgery addiction (*r* = 0.376, *p* < 0.001; moderate correlation). Insecure attachment was significantly and positively correlated with self-alienation (*r* = 0.591, *p* < 0.001; strong correlation). Insecure attachment was significantly and positively correlated with cosmetic surgery addiction (*r* = 0.372, *p* < 0.001; moderate correlation). Self-alienation was significantly and positively correlated with cosmetic surgery addiction (*r* = 0.394, *p* < 0.001; moderate correlation). Correlation strengths were interpreted based on Cohen (65) guidelines: |r| < 0.3 = weak/small; 0.3–0.5 = moderate/medium; >0.5 = strong/large.

### 3.4 Chain mediation analysis of insecure attachment and self-alienation

To further examine the indirect effects of insecure attachment and self-alienation on the relationship between adverse childhood experiences and cosmetic surgery addiction, a bias-corrected bootstrapping method (5,000 samples, 95% confidence interval) was employed, as shown in Table 4. The results indicated: Adverse childhood experiences significantly predicted insecure attachment (*β* = 0.3846, *p* < 0.001, 95% CI = [0.322, 0.448]). Adverse childhood experiences significantly predicted self-alienation (*β* = 0.325, *p* < 0.001, 95% CI = [0.271, 0.381]). Adverse childhood experiences significantly predicted cosmetic surgery addiction (*β* = 0.194, *p* < 0.001, 95% CI = [0.107, 0.281]). Insecure attachment significantly predicted self-alienation (*β* = 0.413, *p* < 0.001, 95% CI = [0.350, 0.475]). Insecure attachment significantly predicted cosmetic surgery addiction (*β* = 0.206, *p* < 0.001, 95% CI = [0.105, 0.308]). Self-alienation significantly predicted cosmetic surgery addiction (*β* = 0.202, *p* < 0.001, 95% CI = [0.087, 0.317]).

**Table 4:** Regression model coefficients for the influence of adverse childhood experiences of cosmetic surgery patients on cosmetic surgery addiction.

Model	Predictor variable	Outcome variable	R	R^2^	F	β	t	LLCI	ULCI
Model 1	Adverse childhood experiences	Insecure attachment	0.439	0.193	143.783***	0.385	11.991***	0.322	0.448
Model 2	Adverse childhood experiences	Self-alienation	0.688	0.474	270.061***	0.325	11.656***	0.271	0.381
	Insecure attachment					0.413	12.939***	0.350	0.475
Model 3	Adverse childhood experiences	Cosmetic surgery addiction	0.459	0.210	53.155***	0.194	4.394***	0.107	0.281
	Insecure attachment					0.206	3.997***	0.105	0.308
	Self-alienation					0.202	3.459***	0.087	0.317

R, Multiple correlation coefficient; R^2^, Coefficient of determination; F, F-statistic; β, Standardized regression coefficient; t, t-statistic; LLCI, Lower limit of 95% confidence interval; ULCI, Upper limit of 95% confidence interval. ***p < 0.001(indicating statistical significance at the 0.1% level).

**Figure 2:**
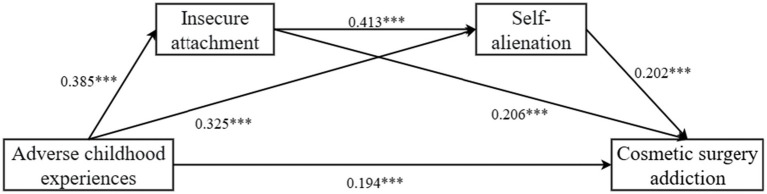
The path coefficient results of the chain mediation of insecure attachment and self-alienation (*p < 0.05; **p < 0.01; ***p < 0.001).

Total and Mediation Effects. As shown in Figure 2 and Table 5, the total effect of adverse childhood experiences on cosmetic surgery addiction was 0.372. Of this, 0.177 was mediated through insecure attachment and self-alienation. Specifically: The indirect effect of adverse childhood experiences → insecure attachment → cosmetic surgery addiction was significant (*β* = 0.079, SE = 0.036, 95% CI = [0.021, 0.152]). The indirect effect of adverse childhood experiences → self-alienation → cosmetic surgery addiction was significant (*β* = 0.066, SE = 0.031, 95% CI = [0.011, 0.133]). The indirect effect of adverse childhood experiences → insecure attachment → self-alienation → cosmetic surgery addiction was significant (*β* = 0.033, SE = 0.013, 95% CI = [0.006, 0.058]). These findings supported Hypotheses H1, H2, H3, and H4.

**Table 5:** Decomposition of the pathways and effects of adverse childhood experiences of cosmetic surgery patients on cosmetic surgery addiction.

Type	Effect size	SE	LLCI	ULCI	Proportion of effect
Total effect	0.372	0.037	0.298	0.445	100%
Direct effect	0.194	0.044	0.108	0.281	52.15%
Ind1	0.079	0.036	0.021	0.152	21.24%
Ind2	0.066	0.031	0.011	0.133	17.74%
Ind3	0.033	0.013	0.006	0.058	8.87%

SE, Standard error; LLCI, Lower limit of 95% confidence interval; ULCI, Upper limit of 95% confidence interval. Ind1, Adverse childhood experiences—Insecure attachment—Cosmetic surgery addiction; Ind 2, Adverse childhood experiences—Self-alienation—Cosmetic surgery addiction; Ind3, Adverse childhood experiences—Insecure attachment—Self-alienation—Cosmetic surgery addiction.

## 4 Discussion

### 4.1 Theoretical implications

#### 4.1.1 The relationship between adverse childhood experiences and cosmetic surgery addiction in cosmetic surgery patients

A significant positive correlation was found between adverse childhood experiences and cosmetic surgery addiction among cosmetic surgery patients. Specifically, individuals who experienced neglect, abuse, or chaotic family environments during childhood were more likely to develop dependencies on cosmetic surgeries. This finding extends the traditional framework of childhood trauma’s impact on adult mental health (66, 67). Previous studies have shown that adverse childhood experiences may lead to a range of psychopathological behaviors by affecting an individual’s self-concept and emotional regulation abilities (41). However, this study further clarifies that cosmetic surgery addiction, as a specific form of behavioral addiction, may stem from unmet psychological needs resulting from childhood trauma. Cosmetic surgery may be perceived by patients as a compensatory mechanism to repair the loss of self-worth caused by adverse childhood experiences. This theoretical contribution not only enriches the scope of childhood trauma research but also provides a new perspective for understanding the psychological roots of cosmetic surgery addiction.

Ecological systems theory emphasizes the influence of microsystems (e.g., family environment) on individual development (68). Adverse childhood experiences, as a core component of the microsystem, may disrupt an individual’s psychological sense of safety, prompting them in adulthood to seek external means of rebuilding self-identity (69). This process suggests that cosmetic surgery addiction is not only an externalization of psychological issues but also reflects the long-term consequences of failing to establish healthy coping mechanisms during childhood. This study empirically validated this theoretical hypothesis, demonstrating that adverse childhood experiences indirectly influences adult behavioral choices by shaping psychological structures. This theoretical contribution deepens the understanding of cosmetic surgery addiction as a complex psychological phenomenon and suggests that future research should focus more on the long-term impact of childhood experiences on behavioral tendencies.

Although adverse childhood experiences is typically viewed as a negative factor, this study revealed that some cosmetic surgery patients attempt to redefine themselves through repeated cosmetic procedures, exhibiting a distorted form of growth. This behavior, though pathological, may be perceived by patients as a means of self-repair. This phenomenon suggests that post-traumatic growth theory needs further expansion to include individuals who cope with trauma in maladaptive ways.

#### 4.1.2 The mediating role of insecure attachment between adverse childhood experiences and cosmetic surgery addiction

Attachment theory posits that caregiver relationships during childhood shape an individual’s attachment style, while insecure attachment may lead to sustained anxiety about intimacy and self-worth in adulthood (70). This study found that adverse childhood experiences increased the risk of cosmetic surgery addiction by disrupting the formation of secure attachment. Individuals with insecure attachment often exhibit excessive dependence on external evaluations (71), and cosmetic surgery may serve as an externalized self-regulation strategy to alleviate psychological distress caused by insecurity. The revelation of this mediating mechanism not only validates the applicability of attachment theory in the domain of behavioral addiction but also provides a new theoretical framework for understanding the psychological motivations behind cosmetic surgery addiction.

This study also explores the potential integration of attachment theory with self-objectification theory (72, 73). Individuals with insecure attachment may be more susceptible to the internalization of societal beauty standards due to a lack of inner security (74), leading them to view cosmetic surgery as a primary means of enhancing self-worth. The study reveals that insecure attachment is not only a direct consequence of adverse childhood experiences but may also amplify negative body image perceptions (32, 75), indirectly contributing to cosmetic surgery addiction. This integration of theories enriches the explanatory framework of attachment theory and provides direction for future research on how to combine multiple theoretical models to explain complex psychological phenomena.

#### 4.1.3 The chain mediation of insecure attachment and self-alienation in the relationship between adverse childhood experiences and cosmetic surgery addiction

This study further revealed the chain mediating roles of insecure attachment and self-alienation in the relationship between adverse childhood experiences and cosmetic surgery addiction, providing a more complex theoretical model for understanding the psychological mechanisms of cosmetic surgery addiction. The study found that adverse childhood experiences shapes insecure attachment, which in turn exacerbates feelings of self-alienation (76, 77). These feelings may drive individuals to seek external self-reconstruction through cosmetic surgery (78). The proposal of this chain mediation mechanism deepens the understanding of the multiple psychological pathways involved in cosmetic surgery addiction and offers psychology a novel theoretical perspective: childhood trauma may gradually evolve into specific behavioral addictions through multilayered psychological processes.

This finding expands the application of self-alienation theory in addiction research (79). Self-alienation is typically considered a core concept in existential psychology (80, 81), but its role in addictive behaviors has not been fully explored. This study empirically demonstrates that self-alienation is not only an indirect consequence of adverse childhood experiences but also plays a critical bridging role between insecure attachment and cosmetic surgery addiction. The repetitive nature of cosmetic surgery may reflect patients’ efforts to bridge their sense of self-alienation by altering their appearance (78), though these efforts are often futile, leading to a vicious cycle of addiction. This discovery provides new scenarios for the application of self-alienation theory and offers theoretical support for intervention studies on cosmetic surgery addiction, suggesting that interventions should focus on the process of self-integration.

### 4.2 Practical implications

#### 4.2.1 Adverse childhood experiences and cosmetic surgery addiction

This study found a significant positive correlation between adverse childhood experiences and cosmetic surgery addiction, providing a key intervention point for mental health professionals. In clinical practice, psychologists and plastic surgeons should systematically screen for patients’ childhood experiences, particularly histories of emotional neglect or physical abuse, during the preoperative assessment. These experiences may lead to distorted self-image perceptions, prompting patients to seek psychological compensation through repeated cosmetic surgeries (82). Based on these findings, it is recommended that medical institutions incorporate standardized childhood experience assessment tools into their preoperative consultation processes to identify high-risk patients and provide psychological interventions rather than relying solely on surgical solutions. Such early identification and intervention strategies could effectively reduce the risk of cosmetic surgery addiction and minimize the physical and psychological harm caused by repeated surgeries.

Plastic surgeons, psychologists, and social workers should collaborate to develop comprehensive intervention plans. For instance, cognitive-behavioral therapy (CBT) could help patients rebuild healthy self-perceptions and alleviate the long-term psychological effects of childhood trauma. Additionally, medical institutions could partner with community organizations to launch mental health education programs for survivors of adverse childhood experiences, raising public awareness of the long-term effects of childhood trauma. Such multilevel interventions would not only aid individual recovery but also reduce the societal incidence of cosmetic surgery addiction.

#### 4.2.2 The mediating role of insecure attachment

Insecure attachment, as a mediator between adverse childhood experiences and cosmetic surgery addiction, provides a new perspective for clinical interventions. In practice, psychotherapists treating cosmetic surgery addiction should focus on assessing patients’ attachment patterns. Using standardized attachment measurement tools can help identify patients’ specific types of insecure attachment, providing a basis for individualized treatment. For example, patients with anxious attachment may benefit from attachment-based therapies that establish a safe relational environment, reducing their excessive focus on appearance and sensitivity to others’ evaluations.

Furthermore, plastic surgeons can collaborate with mental health experts during preoperative consultations to assess patients’ insecure attachment characteristics. For patients exhibiting high levels of insecure attachment, short-term psychological interventions may be recommended to improve their relational patterns and self-worth, thereby reducing their reliance on cosmetic surgery. Such comprehensive interventions could reduce unnecessary surgeries and enhance patients’ psychological well-being. Medical institutions could also develop psychological education programs for cosmetic surgery patients, focusing on attachment theory and self-acceptance training, to help patients understand the deeper psychological roots of their motivations for cosmetic surgery and make more rational decisions preoperatively.

#### 4.2.3 The mediating role of self-alienation

The mediating role of self-alienation in the relationship between adverse childhood experiences and cosmetic surgery addiction has practical implications, particularly in understanding its role in identifying high-risk populations. Clinicians can better recognize individuals at risk of cosmetic surgery addiction due to childhood trauma by understanding the mediating role of self-alienation, enabling early intervention. When developing preventive programs for cosmetic surgery addiction, special attention should be paid to helping individuals establish healthy self-perception and emotional regulation skills. For example, schools could offer mental health education courses to help students better cope with adverse early-life experiences, avoiding the formation of self-alienation as a psychological defense mechanism.

Recognizing the mediating role of self-alienation in cosmetic surgery addiction can provide more targeted strategies for psychological treatments and social interventions. For instance, psychotherapists could use psychodynamic therapy or psychoanalysis to help patients explore and process childhood trauma, reducing the degree of self-alienation and thereby lowering the risk of cosmetic surgery addiction. Additionally, social workers could implement community psychological support projects to assist individuals in self-alienation states caused by adverse childhood experiences, providing them with necessary psychological support and resources to reduce their reliance on cosmetic behaviors.

### 4.3 Limitations and future research directions

One limitation of this study is its cross-sectional design, which restricts in-depth inference of causal relationships between adverse childhood experiences, insecure attachment, self-alienation, and cosmetic surgery addiction. Although structural equation modeling and mediation analysis provided significant correlational evidence, cross-sectional data cannot clearly distinguish the temporal sequence and long-term effects among variables. For example, adverse childhood experiences may indirectly influence cosmetic surgery addiction through other unmeasured psychological mechanisms (e.g., post-traumatic stress disorder or anxiety disorders), which this study did not capture at a single time point. Additionally, the sample was primarily drawn from four tertiary class A hospitals in Sichuan, China, limiting the generalizability of the results due to regional and cultural factors. Different cultures have significantly different standards of beauty and acceptance of cosmetic surgery, such as Western cultures emphasizing individualism, which may result in different psychological mechanisms for cosmetic motivations compared to Eastern cultural contexts. Future studies should adopt longitudinal designs to track the long-term impact of adverse childhood experiences on cosmetic surgery addiction and include multicultural samples to enhance external validity. Furthermore, incorporating qualitative research methods (e.g., in-depth interviews) could provide a more comprehensive understanding of the psychological motivations and cultural factors behind cosmetic surgery addiction, compensating for the limitations of quantitative research.

Another limitation is the reliance on self-report questionnaires to measure adverse childhood experiences, insecure attachment, self-alienation, and cosmetic surgery addiction, which introduces potential subjective biases and recall errors. For instance, the adverse childhood experiences scale requires participants to recall early-life experiences, which may be influenced by memory distortions or social desirability effects, potentially reducing data accuracy. Moreover, while the cosmetic surgery addiction scale underwent cultural adaptation validation, its application in China remains preliminary, and its reliability and validity may be influenced by cultural differences. Future research should combine multisource data (e.g., clinical interviews, family member reports, or medical records) to improve measurement reliability. Additionally, this study did not fully account for potential confounding variables, such as socioeconomic status, media exposure, or peer pressure, which may play regulatory roles in the relationship between adverse childhood experiences and cosmetic surgery addiction. For example, individuals with higher socioeconomic status may be more likely to develop cosmetic surgery addiction due to greater financial capacity. Future studies should include these confounding variables and employ more complex statistical models to further elucidate the mechanisms of cosmetic surgery addiction. Developing cosmetic surgery addiction scales tailored to Chinese cultural contexts would also enhance the credibility and cultural applicability of future research.

Finally, the distribution of gender, age, and surgical sites in the sample may have potential implications for the results. For instance, female patients or those undergoing facial surgeries may exhibit stronger addictive tendencies due to societal pressure regarding appearance. Future studies could conduct subgroup analyses by gender, age, and type of surgery to reveal the heterogeneity of the relationship between adverse childhood experiences and cosmetic surgery addiction in different populations. Additionally, integrating neuroimaging techniques (e.g., fMRI) to explore the effects of adverse childhood experiences, insecure attachment, and self-alienation on the brain’s reward system could provide deeper insights into the neural mechanisms of cosmetic surgery addiction. These multimodal approaches would help construct more comprehensive theoretical models and provide scientific evidence for precise interventions.

## 5 Conclusion

This study empirically demonstrated that adverse childhood experiences significantly predict cosmetic surgery addiction via the mediating roles of insecure attachment and self-alienation. These mechanisms highlight how childhood trauma disrupts emotional connections and self-integration, driving compensatory addictive behaviors in adulthood. Practically, the findings advocate for trauma-informed assessments and interventions, such as cognitive-behavioral therapy, in cosmetic surgery care to mitigate risks and promote psychological well-being. Future longitudinal and multicultural research is needed to validate causality and explore moderating factors.

## Data Availability

The raw data supporting the conclusions of this article will be made available by the authors, without undue reservation.
