# Supplementary material for: The impact of adverse childhood experiences on cosmetic surgery addiction in cosmetic surgery patients
Source: Front Public Health. 2025 Sep 25;13:1682796. doi: 10.3389/fpubh.2025.1682796 (PMC12507770; doi:10.3389/fpubh.2025.1682796)
Supplement: Supplementary file 1 [file Data_Sheet_1.docx]

Appendix

| Scales | Measurement Items (original text) | Measurement Items (Chinese text) | Source of scale |
| --- | --- | --- | --- |
| Cosmetic Surgery Addiction Scale | 나는 계획했던 것보다 더 많은 돈을 성형수술비로 쓴 적이 있다. | 我花了比整容计划更多的钱做整形手术。 | Lim (2008) |
|  | 나는 성형 수술을 한 후 다른 곳을 또 하고 싶은 마음이 든다. | 整形手术后，我想再去别的地方。 |  |
|  | 나는 성형 수술을 한 번 해보고 나서 성형수술에 대한 욕구가 더 커졌다. | 我做过一次整形手术后，对整形手术的欲望更大了。 |  |
|  | 나는 성형 수술을 한 부위가 맘에 들지 않는다면 몇 번이고 재수술을 할 의향이 있다. | 如果对整形手术的部位不满意，我愿意再做几次手术。 |  |
|  | 나는 성형수술을 여기서 그만둔다면 희망이 사라질 것이다. | 如果整形手术就此结束，我就没有希望了。 |  |
|  | 만약 앞으로 성형수술을 할 수 없게 된다면 견디기 힘들 것이다 | 如果以后不能做整形手术，那就难受了。 |  |
|  | 성형 수술에 대한 나의 관심이 가족이나 대인간에 긴장을 조성할 때가 있다. | 我对整形手术的关心有时会造成家人或大人之间的紧张。 |  |
|  | 나는 다른 목적으로 써야 할 돈을 성형 수술을 하는 데 쓴 적이 있다. | 我把应该用于其他目的的钱用在了整形手术上。 |  |
|  | 나는 성형수술비 마련을 위해 무리하게 돈을 벌어 본 적이 있다. | 我为了筹集整形手术费，赚过不少钱。 |  |
|  | 나는 성형수술 때문에 학업이나 업무에 지장을 초래한 적이 있다 | 我曾经因为整形手术而影响学业或工作。 |  |
|  | 나는 주변 사람들로부터 성형 수술을 많이 한다는 지적을 받는다. | 周围的人指责我做了很多整容手术。 |  |
|  | 나는 성형 수술로 인해 정작 써야할 일(생활비, 학비 등)에 돈을 쓰지 못한 적이 있다. | 我曾经因为整容手术，没能把钱花在真正该花的事情(生活费、学费等)上。 |  |
|  | 성형수술은 자신의 외모 결함을 치유해주고 자기만족감을 준다. | 整形手术可以治愈自己的外貌缺陷，给自己带来满足感。 |  |
|  | 만일 내가 성형을 해서 텔레비전이나 영화에 나오는 잘생기고 예쁜 연예인 같은 외모를 갖게 된다면 성형수술을 할 의향이 있다. | 如果我整容后，长得像电视或电影里的英俊帅气的艺人一样，我愿意做整容手术。 |  |
|  | 성형수술은 내 성격을 긍정적으로 변화시킬 수 있을 것이다. | 整形手术可以使我的性格变得积极。 |  |
|  | 나는 성형수술을 하면 지금보다 더 행복해 질 것이라고 생각한다. | 我想做整形手术会比现在更幸福。 |  |
|  | 나는 성형수술을 하고 나면 사회적 성공에 보다 가까워질 수 있을 것이다. | 我做了整形手术后，会更接近社会的成功。 |  |
|  | 성형수술을 통해 나의 자신감을 더욱 향상 하리라 믿는다. | 相信通过整形手术能进一步提高我的自信心。 |  |
|  | 나는 주기적으로 성형외과에서 상담을 받곤 한다. | 我定期到整形外科接受咨询。 |  |
|  | 성형 수술을 위해 시간과 돈을 투자하는 것은 아깝지가 않다. | 为整形手术投入时间和金钱并不可惜。 |  |
|  | 나는 맘에 들지 않는 신체부위가 있다고 느끼면 고치기 전까지는 그에 대한 생각을 떨쳐버릴 수 없다. | 我只要感到有不满意的身体部位，在治好之前就无法摆脱对他的想法。 |  |
|  | 나는 성형 수술 후 예뻐질 내 모습을 기대하면서 평소 괴로운 생각을 잊는다. | 她期待着整形手术后自己变漂亮的样子，却忘记了平时痛苦的想法。 |  |
|  | 나는 현재 생활이 무기력하고 목표가 없을 때 성형수술을 삶의 목표로 삼고자 하거나 그랬던 적이 있다. | 我曾经想过，当我现在生活无力，没有目标的时候，就把整形手术作为生活的目标。 |  |
|  | 나는 다른 일을 하는 것보다 성형에 관한 정보를 얻는데 많은 시간을 소비한다. | 比起做其他工作，我花更多的时间获取整形相关的信息。 |  |
| Insecure Attachment Scale | I'm afraid that I will lose my partner's love. | 我害怕失去伴侣的爱。 | Fraley et al. (2000) |
|  | I often worry that my partner will not want to stay with me. | 我经常担心我的伴侣不想和我在一起。 |  |
|  | I often worry that my partner doesn't really love me. | 我经常担心我的伴侣不是真的爱我。 |  |
|  | I worry that romantic partners won't care about me as much as I care about them. | 我担心恋人不会像我关心他们那样关心我。 |  |
|  | I often wish that my partner's feelings for me were as strong as my feelings for him or her. | 我经常希望我的伴侣对我的感情和我对他或她的感情一样强烈。 |  |
|  | I worry a lot about my relationships. | 我很担心我的人际关系。 |  |
|  | When my partner is out of sight, I worry that he or she might become interested in someone else. | 当我的伴侣离开我的视线时，我担心他或她可能会对别人感兴趣。 |  |
|  | When I show my feelings for romantic partners, I'm afraid they will not feel the same about me. | 当我表达对恋人的感情时，我害怕他们对我的感觉不一样。 |  |
|  | I rarely worry about my partner leaving me. | 我很少担心我的伴侣会离开我。 |  |
|  | My romantic partner makes me doubt myself. | 我的恋人让我怀疑自己。 |  |
|  | I do not often worry about being abandoned. | 我不常担心被抛弃。 |  |
|  | I find that my partner(s) don't want to get as close as I would like. | 我发现我的伴侣并不想像我希望的那样亲近。 |  |
|  | Sometimes romantic partners change their feelings about me for no apparent reason. | 有时候浪漫的伴侣会莫名其妙地改变对我的感觉。 |  |
|  | My desire to be very close sometimes scares people away. | 我想要亲近的欲望有时会把别人吓跑。 |  |
|  | I'm afraid that once a romantic partner gets to know me, he or she won't like who I really am. | 我害怕一旦一个浪漫的伴侣了解了我，他或她就不会喜欢真正的我。 |  |
|  | It makes me mad that I don't get the affection and support I need from my partner. | 我不能从我的伴侣那里得到我需要的爱和支持，这让我很生气。 |  |
|  | I worry that I won't measure up to other people. | 我担心自己达不到别人的标准。 |  |
|  | My partner only seems to notice me when I'm angry. | 我的伴侣似乎只有在我生气的时候才会注意到我。 |  |
|  | I prefer not to show a partner how I feel deep down. | 我不想让伴侣看到我内心深处的感受。 |  |
|  | I feel comfortable sharing my private thoughts and feelings with my partner. | 与我的伴侣分享我的私人想法和感受，我感到很舒服。 |  |
|  | I find it difficult to allow myself to depend on romantic partners. | 我发现很难让自己依赖恋人。 |  |
|  | I am very comfortable being close to romantic partners.. | 我和恋人在一起很舒服。 |  |
|  | I don't feel comfortable opening up to romantic partners. | 我对恋人敞开心扉感到不自在。 |  |
|  | I prefer not to be too close to romantic partners. | 我不喜欢和恋人走得太近。 |  |
|  | I get uncomfortable when a romantic partner wants to be very close. | 当一个浪漫的伴侣想要非常接近时，我会感到不舒服。 |  |
|  | I find it relatively easy to get close to my partner. | 我发现相对容易接近我的伴侣。 |  |
|  | It's not difficult for me to get close to my partner. | 对我来说，接近我的伴侣并不难。 |  |
|  | I usually discuss my problems and concerns with my partner. | 我通常和我的伴侣讨论我的问题和担忧。 |  |
|  | It helps to turn to my romantic partner in times of need. | 在需要的时候向我的爱人求助是很有帮助的。 |  |
|  | I tell my partner just about everything. | 我什么都跟我的搭档说。 |  |
|  | I talk things over with my partner. | 我和我的搭档商量事情。 |  |
|  | I am nervous when partners get too close to me. | 当伴侣离我太近时，我很紧张。 |  |
|  | I feel comfortable depending on romantic partners. | 我觉得依赖恋人很舒服。 |  |
|  | I find it easy to depend on romantic partners. | 我觉得依赖恋人很舒服。 |  |
|  | It's easy for me to be affectionate with my partner. | 对我来说，对我的伴侣充满深情是很容易的。 |  |
|  | My partner really understands me and my needs. | 我的伴侣真正理解我和我的需求。 |  |
| Self- Alienation Scale | I often feel as if things are not real. | 我经常觉得事情好像不真实。 | Nishiyama et al. (2022) |
|  | Almost every day something happens to frighten me. | 几乎每天都有让我害怕的事情发生。 |  |
|  | I hear strange things when I am alone. | 我一个人的时候会听到奇怪的声音。 |  |
|  | Bad words, often terrible words, come into my mind and I cannot get rid of them. | 坏的词，通常是可怕的词，进入我的脑海，我无法摆脱它们。 |  |
|  | Often, even though everything is going fine for me, I feel that I don't care about anything. | 通常，即使我一切都很好，我也觉得我什么都不在乎。 |  |
|  | People often disappoint me. | 人们经常让我失望。 |  |
|  | I have difficulty in starting to do things. | 我很难开始做事情。 |  |
|  | There is something wrong with my mind. | 我的脑子有问题。 |  |
|  | Sometimes I am sure that other people can tell what I am thinking. | 有时我确信别人能看出我在想什么。 |  |
|  | I sometimes feel that I am about to go to pieces. | 我有时觉得自己快要崩溃了。 |  |
|  | I have had blank spells in which my activities were interrupted and I did not know what was going on around me. | 我曾经有过一段空白期，我的活动被打断，我不知道周围发生了什么。 |  |
|  | I am so touchy on some subjects that I can't talk about them. | 我对一些话题很敏感，所以不能谈论它们。 |  |
|  | People say insulting and vulgar things about me. | 人们对我说一些侮辱和粗俗的话。 |  |
|  | I have more trouble concentrating than others seem to have. | 我比别人更难以集中注意力。 |  |
|  | At times I hear so well it bothers me | 有时我听得很清楚，这让我很烦恼。 |  |
|  | Sometimes I become so excited that I find it hard to get to sleep | 有时我太兴奋了，以至于很难入睡。 |  |
|  | I sometimes keep on at a thing until others lose their patience with me | 我有时会坚持做一件事，直到别人对我失去耐心。 |  |
|  | There are persons who are trying to steal my thoughts and ideas | 有些人试图窃取我的思想和想法。 |  |
|  | I am sure I get a raw deal from life. | 我确信我从生活中得到了不公平的待遇。 |  |
|  | My people treat me more like a child than a grown-up. | 我的人待我更像个孩子，而不是大人。 |  |
|  | I have sometimes stayed away from another person because I feared doing or saying something that I might regret afterwards. | 我有时会远离另一个人，因为我害怕做或说一些事后可能会后悔的话。 |  |
|  | I have been disappointed in love. | 我对爱情失望过。 |  |
|  | I commonly hear voices without knowing where they come from. | 我经常听到不知道从哪里来的声音。 |  |
|  | Most of the time I feel blue. | 大多数时候我都感到忧郁。 |  |
|  | Whenever possible I avoid being in a crowd. | 只要有可能，我就避免待在人群中。 |  |
|  | Someone has control over my mind. | 有人控制了我的思想。 |  |
|  | Often I feel as if there were a tight band about my head. | 我常常觉得好像有一条紧箍在我的头上。 |  |
| Adverse Childhood Experiences Scale | Do you agree that when you were a child, your partner or other adult often swore at you, insulted you, belisted you, humiliated you, or acted in ways that made you fear that you would be physically harmed? | 您是否同意在您儿童时期，您的伙伴或其他成年人经常地咒骂您，侮辱您，贬低您，羞辱您，或做出让您害怕自己会受到身体伤害的行为？ | Finkelhor et al. (2015) |
|  | Do you agree that when you were a child, your partner or other adult often or frequently pushed, grabbed, slapped or threw things at you or hit you so hard that you had cuts or injuries? | 您是否同意在您儿童时期，您的伙伴或其他成年人经常或经常地推、抓、扇或扔东西给您或者打得太狠，弄得您身上有伤痕或者受伤了？ |  |
|  | Do you agree that during your childhood, you experienced an adult at least 5 years older than you touching or caressing you, or did you touch their body in a sexual manner or attempt or actually engage in oral, anal or vaginal sex with you? | 您是否同意在您儿童时期，您经历过一个比您大至少5岁的成年人触摸或爱抚您，或者您是否以性的方式触摸过他们的身体或尝试或实际与您进行口交、肛交或阴道性交？ |  |
|  | Do you agree that as a child, you often felt that no one in your family loved you, thought you were important or special or that your family did not take care of each other, feel close to each other, or support each other? | 您是否同意在您儿童时期，您经常觉得，您的家人没有人爱您，认为您很重要或很特别或者您的家人没有互相照顾，感觉彼此亲近，或相互支持？ |  |
|  | Do you agree that as a child, you often felt that you did not have enough to eat, had to wear dirty clothes, had no one to protect you or that your partner was drunk or on drugs and could not look after you or take you to the doctor when you needed it? | 您是否同意在您儿童时期，您经常感到您没有足够的食物，不得不穿脏衣服，没有人保护您或者您的伴侣喝醉了或者嗑药了，不能照顾您或者在您需要的时候带您去看医生？ |  |
|  | Do you agree that when you were a child, you lost your friendliest partner because of abandonment or other reasons? | 您是否同意在您儿童时期，您曾因被遗弃或其他原因失去最友好的伙伴？ |  |
|  | Do you agree that during your childhood, your mother or stepmother often or often pushed you, scratched you, slapped you, or someone threw something at you or sometimes, often kicked, bit, punched you, or hit you with something hard, or you were hit multiple times or threatened with a gun or knife? | 您是否同意在您儿童时期，您的母亲或继母经常或经常推您、抓您、扇您耳光，或者有人朝您扔东西或有时，经常踢，咬，用拳头打，或用硬东西打或您、曾多次被击中或被枪或刀威胁过？ |  |
|  | Do you agree that during your childhood, you lived with an alcoholic or alcoholic, or street drug user? | 您是否同意在您儿童时期，您曾与嗜酒者或酗酒者，或吸食街头毒品的人同住？ |  |
|  | Do you agree that during your childhood, you had a family member who suffered from depression or mental illness and a family member who attempted suicide? | 您是否同意在您儿童时期，您有家庭成员患有抑郁症或精神疾病，家庭成员企图自杀？ |  |
|  | Do you agree that any of your family members went to prison during your childhood? | 您是否同意在您儿童时期，您有家庭成员进监狱了吗？ |  |
|  | Do you agree that during your childhood, other children, including siblings, often hit, threatened, bullied or insulted you? | 您是否同意在您儿童时期，其他孩子包括兄弟姐妹经常打您、威胁您、欺负您或侮辱您？ |  |
|  | Do you agree that as a child, you often felt lonely, rejected or unliked? | 您是否同意在您儿童时期，您经常感到孤独、被拒绝或没有人喜欢您？ |  |

Reference:

Finkelhor, D., Shattuck, A., Turner, H., & Hamby, S. (2015). A revised inventory of adverse childhood experiences. *Child abuse & neglect*, *48*, 13-21.

Fraley, R. C., Waller, N. G., & Brennan, K. A. (2000). An item response theory analysis of self-report measures of adult attachment. *Journal of Personality and Social Psychology*, *78*(2), 350.

Lim, I. (2008). Preliminary study for development of aesthetic plastic surgery addiction scale. master's thesis. *Chungbuk National University, Cheongju*.

Nishiyama, S., Kurachi, M., Higuchi, Y., Takahashi, T., Sasabayashi, D., Mizukami, Y., & Suzuki, M. (2022). Development and validation of a scale of self-alienation-related attributes for the early diagnosis of schizophrenia. *Journal of Psychiatric Research*, *147*, 212-220.
